# Experimental investigation on preconditioned rate induced tipping in a thermoacoustic system

**DOI:** 10.1038/s41598-017-05814-y

**Published:** 2017-07-14

**Authors:** J. Tony, S Subarna, K. S. Syamkumar, G. Sudha, S. Akshay, E. A. Gopalakrishnan, E. Surovyatkina, R. I. Sujith

**Affiliations:** 1 Indian Institute of Technology Madras, Chennai, India; 2 Center for Computational Engineering and Networking (CEN), Amrita Vishwa Vidyapeetham, Amrita University, Coimbatore, India; 3 Space Research Institute of Russian Academy of Sciences, Moscow, Russia; 4 Potsdam Institute for Climate Impact Research, Potsdam, Germany

## Abstract

Many systems found in nature are susceptible to tipping, where they can shift from one stable dynamical state to another. This shift in dynamics can be unfavorable in systems found in various fields ranging from ecology to finance. Hence, it is important to identify the factors that can lead to tipping in a physical system. Tipping can mainly be brought about by a change in parameter or due to the influence of external fluctuations. Further, the rate at which the parameter is varied also determines the final state that the system attains. Here, we show preconditioned rate induced tipping in experiments and in a theoretical model of a thermoacoustic system. We provide a specific initial condition (preconditioning) and vary the parameter at a rate higher than a critical rate to observe tipping. We find that the critical rate is a function of the initial condition. Our study is highly relevant because the parameters that dictate the asymptotic behavior of many physical systems are temporally dynamic.

## Introduction

Tipping^1–8^ in a dynamical system can happen either due to a bifurcation in the system known as B-tipping or due to the influence of noise known as N-tipping^9^. In B-tipping, the parameter is changed in a static manner, i.e., the parameter is independent of time. At a critical value of the parameter, one dynamical state becomes unstable and the system shifts to a new stable state. The scenario is different in N-tipping where the fluctuations in the system can tip the system to an alternate state. Further, there exists an alternate classification of these bifurcations into safe, explosive and dangerous, depending on the severity of the bifurcation. A detailed description of this classification is provided in the review paper by Thompson & Sieber^10^. In order to identify bifurcations in a system, the control parameter must be varied in a static or quasi-static manner. However, in real situations, the control parameter will be varied as function of time in physical systems. As a result of the change in parameter with time, the dynamics of the system will also depend on the rate at which the parameter is varied.

The transition to an alternate stable state can be delayed when the control parameter is varied at a slow rate^11–16^. The scenario is different when the parameter is varied in a rapid manner, where rate depending tipping or R-tipping^9, 17^ could occur. In certain systems, the system follows the gradually varying quasi-static attractor when the rate at which the parameter is varied is below a critical rate. In contrast, when the parameter is varied above the critical rate, the system will be driven outside the basin of attraction of the quasi-static attractor and will eventually be attracted to a new stable state. The sufficient conditions for a specific form of R-tipping for saddle-node and Hopf bifurcation are detailed in Ashwin *et al*.^9^.

Here, we demonstrate a preconditioned rate induced tipping in laboratory scale experiments and in a theoretical model of a bistable system. The system considered in this study undergoes a subcritical Hopf bifurcation for a suitable variation in its control parameter. In order to show the differences between our study and that of Ashwin *et al*.^9^, we introduce the concept of preconditioned rate induced tipping in the normal form equation of such a system. The normal form for a bistable system that undergoes subcritical Hopf bifurcation can be written in terms of amplitude (*r*) and phase (*θ*) as

1 $$\dot{r}=\mu r+a{r}^{3}-b{r}^{5},\quad \dot{\theta }=1$$

The bistable region gets its name from the presence of two attractors - a stable fixed point and a stable limit cycle in the case of equation (1). These attractors are separated by an unstable limit cycle. As the parameter *μ* is increased, the stable and the unstable limit cycles are born at the fold point whereas the fixed point loses its stability at the Hopf point.

**Figure 1:**
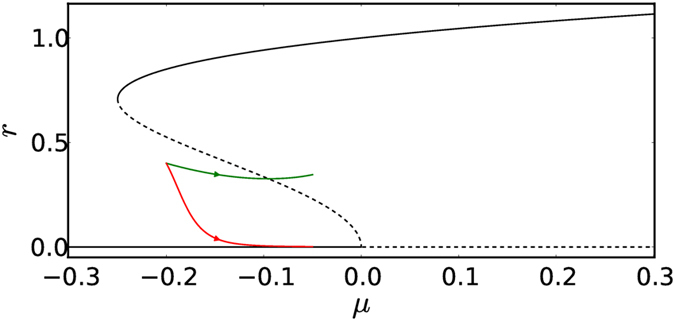
Bifurcation diagram depicting the plot of *r* as a function of *μ* for the system given in equation (1) with *a* = 1 and *b* = 1. The Hopf bifurcation occurs at *μ* = 0 and the fold bifurcation occurs at *μ* = −0.25. The solid black lines represent stable limit cycle while the dashed black lines represent the unstable limit cycle. The red and green lines denote the trajectory followed by the system (equation 1) for the same initial condition (*r*_0_ = 0.4, *μ*_0_ = −0.2) while the parameter is varied at different rates ($\dot{\mu }=c$) till *μ*_*e*_ = −0.05. When the rate $\dot{\mu }=0.003$ is lower than a critical value (*c*_*r*_ = 12.83 × 10^−3^ for this set of parameters), the system decays to the fixed point (red trajectory). The system crosses the unstable limit cycle when the rate of change of parameter $\dot{\mu }=0.02$ is above the critical value (green trajectory).

Starting from the basin of attraction of the fixed point, one has to reach the Hopf point for the system to start displaying limit cycle oscillations, when the control parameter *μ* is changed in a quasi-static manner. We, here, show that, on varying the control parameter fast enough, one can cross over from the basin of attraction of the fixed point to that of the stable limit cycle before reaching the Hopf point (see Fig. 1). Therefore, the system can tip before the actual loss of stability of the fixed point - a phenomenon induced purely due to fast enough variation of the control parameter. In Supplementary Note 1, we demonstrate that for any given initial condition, sufficiently large rates can be found so that the system (equation 1) tips. We also show that for the same initial condition, a small enough rate exists for which the system does not tip. This shows that the rate at which the parameter is varied determines whether the system tips, and it also implies the existence of a critical rate above which tipping occurs.

We now juxtapose our study with that of Ashwin *et al*.^9^ to highlight the differences between the two. Ashwin *et al*.^9^ showed that R-tipping is possible in a system exhibiting subcritical Hopf bifurcation. In their study, however, the bifurcation parameter *μ* is fixed, implying that the radius of the unstable limit cycle and the position of the fixed point relative to the unstable limit cycle remain unaltered. They change a different parameter that leads to the motion of the attractors in some direction, while maintaining their overall structure. They show that if this other parameter is varied fast enough, the system can tip. We, on the other hand, change the bifurcation parameter *μ* at a finite rate to bring about preconditioned rate induced tipping. We consider that tipping has occurred when the system is unable to follow the fixed point (corresponding to the static case) and moves out of its basin boundary (see Fig. 1). In the R-tipping mechanism described in Ashwin *et al*.^9^, the critical rate has the same value for a set of initial conditions. However, in our case, the critical rate associated with tipping is different for each initial condition (More details in the Results section). Therefore, we term the tipping mechanism introduced in this study as preconditioned rate induced tipping. The scenario we consider is very relevant as it is often the parameter *μ* that is changed in many physical systems.

We now explore the possibility of observing preconditioned rate induced tipping in experiments. The experiments are conducted in a thermoacoustic system which has the features of a nonlinear bistable oscillator^18, 19^ (see Methods for details on the experimental setup). A thermoacoustic system is a self-excited system where an unsteady heat source is located in a duct^20^. A positive feedback^21^ established between the heat release rate fluctuations and the sound waves in the duct could result in a transition to limit cycle oscillations. The limit cycle oscillations are characterized by large amplitude fluctuations in pressure and velocity. Such oscillations are encountered in aircraft and rocket engines where combustion is used as a source of power^22^. Tipping in a thermoacoustic system is undesirable because the resulting oscillations can reduce the operational envelope of the system or even result in the failure of the system^22^. Therefore, identifying the factors that result in tipping in a thermoacoustic system has practical relevance. In the specific system we study, the heat source is an electric heater with heater power (*K*) as the control parameter. In addition to experiments, we investigate preconditioned rate induced tipping in a theoretical model^23, 24^.

## Experimental setup

The experimental setup consists of a horizontal duct of length 1 m with square cross-section of dimension 95 mm × 95 mm. An electrically heated wire mesh is the source of heat for this system. A compressor is used to establish the airflow in the duct. One end of the duct is connected to a rectangular chamber while the other end is open to the atmosphere. The rectangular chamber, referred to as decoupler, has a size (1200 mm × 450 mm × 450 mm) much larger than the cross-section of the duct. The decoupler ensures that the fluctuations from the compressor decay before they enters the duct. Thus, we maintain the pressure to be at the ambient value at both the ends of the duct. Loudspeakers are used to establish the initial condition required in experiments. The details related to data acquisition are included in Methods. Additional information on the experimental set-up and the schematic of the set-up can be found in Gopalakrishnan *et al*.^24^. In all the experiments conducted, the volume flow rate was maintained at 150 SLPM and the heater was located at 25 cm from the end connected to the decoupler.

## Theoretical model

The theoretical model used in this study is derived from conservation equations of momentum and energy. We decompose the variables pressure, velocity and density into mean and fluctuating quantities. In the system considered in this study, the amplitude of the fluctuations are small. Therefore, terms involving product of fluctuating quantities are neglected. The effect of mean flow and temperature gradient are also neglected. The derivation of the model is included in Supplementary Note 2. The resulting equations are non-dimensionalized to obtain the following equations.

2 $$\gamma M\frac{\partial u^{\prime} }{\partial t}+\frac{\partial p^{\prime} }{\partial x}=0,$$

3 $$\frac{\partial p^{\prime} }{\partial t}+\gamma M\frac{\partial u^{\prime} }{\partial x}=k[\sqrt{|\frac{1}{3}+u^{\prime} (t-\tau )|}-\sqrt{\frac{1}{3}}]\delta (x-{x}_{f}),$$

Here, *u*′ and *p*′ represent the fluctuations in velocity and pressure above a mean level (*u*_0_ and $\bar{p}$ respectively). *k* is the control parameter which is analogous to the heater power (*K*) in experiments, *τ* is the time delay, *M* is the mean flow Mach number (*u*_0_/*c*_0_), *c*_0_ is the speed of sound in the duct and *x*_*f*_ is the heater location.

The above partial differential equations can be converted to a set of ordinary differential equations using the Galerkin^25, 26^ expansion. The choice of the basis function for this expansion is such that the boundary conditions are satisfied. The variables *u*′ and *p*′ are expressed in terms of the natural acoustic modes of the duct as

4 $$u^{\prime} =\sum _{j=1}^{N}{\eta }_{j}(t)\cos \,j\pi x,\quad p^{\prime} =-\sum _{j=1}^{N}\frac{\gamma M}{j\pi }{\dot{\eta }}_{j}(t)\sin \,j\pi x$$

Here, *N* is the total number of modes, *jπ* is the non-dimensional frequency of the *j* ^th^ mode. ${\dot{\eta }}_{j}$ is the time derivative of *η*_*j*_. The pressure at both ends of the duct is equal to ambient pressure. This implies that the pressure fluctuations at both ends of the duct are zero. We can notice that the boundary conditions *p*′(*x* = 0) = 0 and *p*′(*x* = 1) = 0 are satisfied by the basis functions. Also, note that equation (1) is trivially satisfied by (4). We substitute the expressions for *u*′ and *p*′ in equation (3). After the substitution, we multiply equation (3) by $\sin \,n\pi x$ and integrate along the domain [0, 1]. Through this process, we are projecting the equations along a particular basis function. Thus, we obtain a second order ODE that governs the evolution of *η*_*j*_(*t*).

5 $${\ddot{\eta }}_{j}+2{\zeta }_{j}{\omega }_{j}{\dot{\eta }}_{j}+{\omega }_{j}^{2}{\eta }_{j}=-kj\pi [\sqrt{|\frac{1}{3}+{u}_{f}^{^{\prime} }(t-\tau )|}-\sqrt{\frac{1}{3}}]\sin \,j\pi {x}_{f}$$

where ${u}_{f}^{^{\prime} }(t-\tau )=\sum _{j=1}^{N}\,{\eta }_{j}(t-\tau )\cos \,j\pi {x}_{f}$. We introduce a damping term with damping coefficient ${\zeta }_{j}=\frac{1}{2\pi }[{c}_{1}\frac{{\omega }_{j}}{{\omega }_{1}}+{c}_{2}\sqrt{\frac{{\omega }_{1}}{{\omega }_{j}}}]$, where *c*_1_ and *c*_2_ are constant coefficients, in equation (5). The model shows transition from a non-oscillatory to an oscillatory state via subcritical Hopf bifurcation^18^ for suitable change in the parameter *k*. We normalize *k* to obtain a new parameter $\tilde{k}=k/{k}_{H}-1$, where *k*_*H*_ represents the value of *k* at the Hopf point. We identify $\tilde{k}$ as the control parameter in the model. We find that the value of *k*_*H*_ = 0.62 for the parameters considered in the present study (see Methods summary).

We now list the approximations involved in deriving the model. The heat release rate expression $\tilde{\dot{Q}}^{\prime}$ (equation 21 in Supplementary Note 2) does not capture the exact physical conditions in experiments. The expression models heat transfer from a cylindrical wire which does not perfectly represent the heat transfer from a mesh (the heating element used in physical experiments). Further, we neglected the effect of mean flow in the model. These changes bring in the quantitative differences between the model and experiments.

## Preconditioned rate dependent tipping in a thermoacoustic system

**Figure 2:**
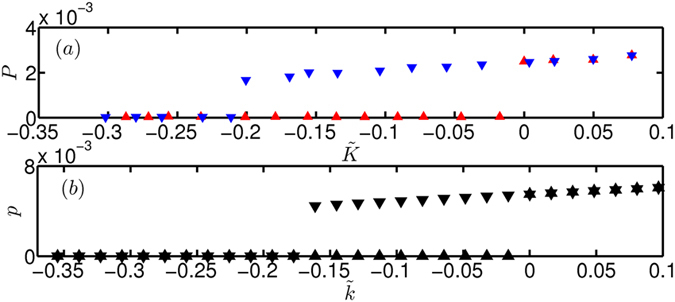
Bifurcation diagram depicting the change in the rms value of acoustic pressure with control parameter obtained from (**a**) experiments and (**b**) theoretical model. In (**a**) experiments, the heater power *K* is changed in a quasi-stationary manner. The red and blue triangles represent the forward and backward paths respectively. The presence of a bistable region and a sudden increase in the amplitude of acoustic pressure at the Hopf point indicate that the system undergoes a subcritical Hopf bifurcation. The parameters at which Hopf and fold bifurcation occurs are $\tilde{K}=0$ and $\tilde{K}=-0.21$ respectively in this particular experiment. In (**b**) the model also, the presence of a bistable region and sudden increase in the amplitude of non-dimensional acoustic pressure at the Hopf point indicate that the system undergoes a subcritical Hopf bifurcation. The parameters at which Hopf and fold bifurcation occurs are ${\tilde{k}}_{H}=0$ and ${\tilde{k}}_{f}=-0.18$ respectively in the model. Note that the model captures the experimental features only qualitatively.

In order to demonstrate preconditioned rate induced tipping, we need to identify the parameter regime where the system is bistable. Therefore, we performed a bifurcation analysis in experiments and in the theoretical model. In experiments, we varied the heater power (*K*) in a quasi-stationary manner and acquired the pressure fluctuations (acoustic pressure). Figure 2a represents the variation of the rms value of non-dimensional acoustic pressure as a function of the normalized heater power $\tilde{K}$ (=*K*/*K*_*H*_ − 1), where *K*_*H*_ = 827.8 W for the bifurcation diagram presented in Fig. 2a. Time is non-dimensionalized with the travel time of sound in the duct, *L*/*c*_0_, where *c*_0_ (≈347 m/s) is the speed of sound corresponding to the ambient temperature (300 K) and *L* is the length of the horizontal duct (1 m). To obtain the bifurcation diagram, we increase the heater power (we refer to it as the forward path) until we reach the Hopf point where the fixed point becomes unstable. We can see from Fig. 2a that the system remains in the non-oscillatory state till $\tilde{K}$ reaches 0 (Hopf point). At this point, the system transits to the stable limit cycle that exists for the same value of the parameter. Then, we decrease the heater power (reverse path) and the system continues to remain on the stable limit cycle. When the fold point ($\tilde{K}=-0.21$) is reached, the limit cycle vanishes and the system moves to the stable fixed point available at the same value of the parameter. Note that the bifurcation diagram presented in Fig. 2a is obtained from a single experiment.

We can observe that the point of transition to and from the oscillatory state are different which marks the presence of a bistable region. The sudden increase in the observable (acoustic pressure) along with the presence of bistable region confirms that the system undergoes a subcritical Hopf bifurcation as it transits from non-oscillatory to oscillatory state. The system is brought back to the non-oscillatory state through a fold bifurcation. Similar to experiments, the static bifurcation diagram from the theoretical model also shows that the system undergoes a subcritical Hopf bifurcation (see Fig. 2b).

Thereafter we proceed to demonstrate the presence of preconditioned rate induced tipping experimentally in our system. The first step is choosing an initial condition (for the control parameter as well as the system state) for beginning the experiment. Towards this purpose, the value of the parameter $\tilde{K}$ is maintained at −0.135. Next, we choose the initial state of the system to be below the unstable limit cycle amplitude, so that it is naturally attracted to the fixed point. Towards this, we establish a finite amplitude perturbation to the system using loudspeakers, and check if the perturbation decays to the non-oscillatory state. Decay in the perturbation would indicate that the system is in the basin of attraction of the fixed point. In experiments, we identified a finite amplitude perturbation which decays when the control parameter is maintained at −0.135 (see Supplementary Notes 3 and Supplementary Fig. 3.1).

**Figure 3:**
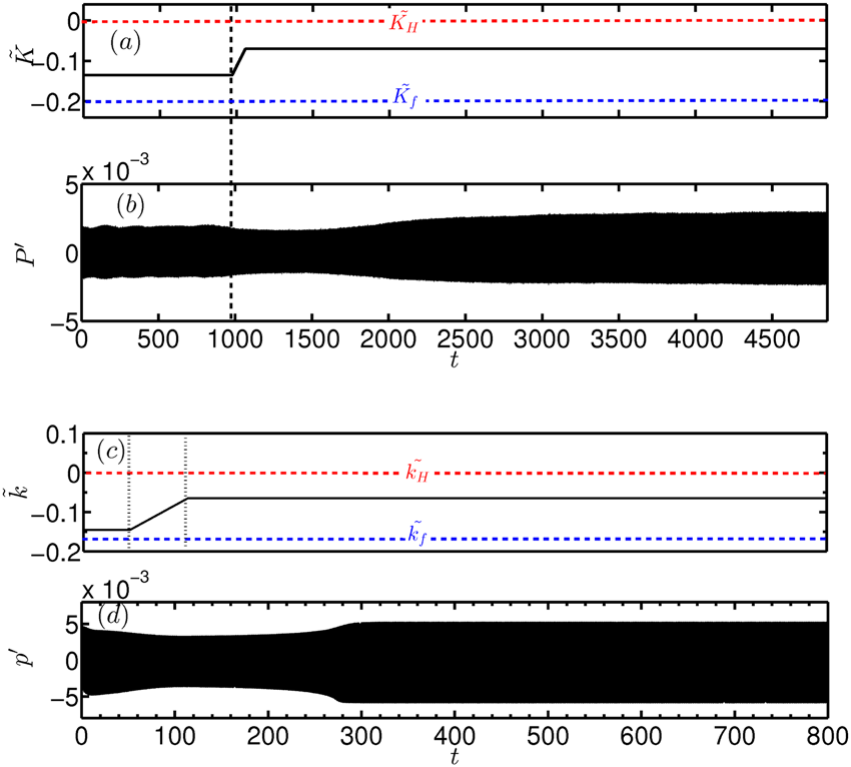
Preconditioned rate induced tipping observed in experiments and in the mathematical model of a thermoacoustic system. (**a**) The variation of heater power with time in experiments. The red and blue horizontal lines denote the values of ${\tilde{K}}_{H}$ (Hopf point) and ${\tilde{K}}_{f}$ (fold point) obtained from a quasi-static bifurcation experiment. The heater power is varied such that the system is within the bistable zone (**b**) The variation of acoustic pressure with time in experiments. The value of the control parameter is maintained at ${\tilde{K}}_{0}=-0.135$ and an initial excitation of amplitude which is within the basin of attraction of the fixed point is provided to the system using loudspeakers, and then the speakers are switched off. The vertical dashed line indicates the time stamp at which the loud speakers are switched off (*t* = 971). Then, the heater power is increased to ${\tilde{K}}_{e}=-0.07$ following $\tilde{K}={\tilde{K}}_{0}+\beta (t-971)$ where *β* = 6.07 × 10^−4^. As a result, the system tips to the oscillatory state. (**c**) The variation of non-dimensional heater power with time in the mathematical model. The red and blue dashed horizontal lines denote the Hopf and fold points. The value of the non-dimensional heater power is maintained at ${\tilde{k}}_{0}=-0.145$ which is slightly greater than the value of $\tilde{k}$ at the fold point up to *t*_0_ = 50. Then the non-dimensional heater power is increased from ${\tilde{k}}_{0}=-0.145$ to ${\tilde{k}}_{e}=-0.064$ according to the relation $\tilde{k}={\tilde{k}}_{0}+\alpha (t-{t}_{0})$, where *α* = 1.29 × 10^−3^. (**d**) The variation of non-dimensional acoustic pressure with time shows that the system tips to limit cycle oscillations after the change in $\tilde{k}$. The parameters involved in the model are included in Methods summary.

Once the initial conditions are established, the loudspeakers are turned off and we proceed to change the heater power fast enough so that the system tips. Caution must be exercised to ensure that tipping is not due to crossing the Hopf point. Towards this purpose, we choose the final value of the parameter $\tilde{K}$ to be −0.07, which is significantly lower than the value of $\tilde{K}$ at the quasi-stationary Hopf point (see Fig. 3a). As mentioned before, the initial perturbation to the system state is provided using loudspeakers, which are switched off at *t* = 971 (*t* is non-dimensional). This is immediately followed up by an increase in the parameter from the initial value of −0.135 to the final value of −0.07 in a time span of ${\rm{\Delta }}t=107$ (in a linear fashion). This change of parameter at a finite rate causes the system to tip to the oscillatory state, as observed from Fig. 3b. We can notice that, after an initial decay in the amplitude, the system eventually portrays large amplitude oscillations.

Further, we have investigated the possibility of establishing preconditioned rate induced tipping in the mathematical model of the system. As in the case of experiments, we choose an initial control parameter value ${\tilde{k}}_{0}$, so that the system is in the bistable region (Fig. 3c). Further, we choose an initial perturbation amplitude that decays when the system is maintained at this parameter (see Supplementary Notes 3 and Supplementary Fig. 3.2). Such a decay ensures that the initial condition provided for the simulation is within the basin of attraction of the fixed point. We then increase the value of the heater power from ${\tilde{k}}_{0}$ to ${\tilde{k}}_{e} < {\tilde{k}}_{H}$ at a particular rate, in a linear fashion. The result of such an increase in the control parameter value is similar to what is observed in the experiments: after an initial decay, the perturbation grows and the system transits to the state of stable limit cycle oscillations (see Fig. 3d). As before, we ensure that the values of the control parameter and the amplitude of the initial perturbation are such that bifurcation induced tipping is ruled out. Thus, the tipping that we observe in the mathematical model can also be classified as preconditioned rate induced tipping.

**Figure 4:**
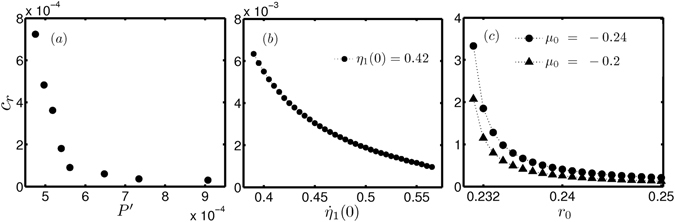
Critical rate required for preconditioned rate induced tipping in (**a**) experiments (**b**) theoretical model and (**c**) normal form equation of subcritical Hopf bifurcation. In (**a**) experiments, we provide an initial periodic perturbation of amplitude *P*′ (below the unstable limit cycle) and vary the parameter from $\tilde{K}=-0.135$ to −0.07. We find the minimum rate (*c*_*r*_) at which the tipping occurs for each initial condition. In (b) theoretical model, the parameter is varied from ${\tilde{k}}_{0}=-0.145$ to ${\tilde{k}}_{e}=-0.064$ following $\tilde{k}={\tilde{k}}_{0}+\alpha (t-{t}_{0})$, where *t*_0_ = 50, for a set of initial conditions to obtain the critical rate. We set *η*_1_(0) = 0.42 and varied ${\dot{\eta }}_{1}(0)$ in the model. In (**c**) the normal form ($\dot{r}=\mu r+a{r}^{3}-b{r}^{5}$, *a* = 1, *b* = 1), the parameter *μ* is varied from *μ*_0_ to a final value *μ*_*e*_ = −0.05. We observe that the critical rate decreases with an increase in *μ*_0_ for the same initial condition. The procedure followed to obtain the critical rates in experiments and model is detailed in Methods.

Next, we obtain an estimate for the critical rate required to observe tipping in experiments, theoretical model and normal form equation of subcritical Hopf bifurcation (Fig. 4). In each of these systems, we provide an initial condition which is within the basin of attraction of the stable fixed point. Then, we vary the parameter from an initial to a final value (within the bistable zone). We find the minimum rate required for the system to undergo tipping for the given initial condition. We repeat this process and estimate the critical rate for various initial conditions. We can see from Fig. 4 that the critical rate for tipping is low if the initial condition is close to the unstable limit cycle. The variation of critical rate with initial condition follows a similar trend in experiments, model and normal form equation.

Thus, in this study, we demonstrate tipping, which occurs as a result of a sudden increase in control parameter, through physical and numerical experiments. We estimate the critical rate required to observe tipping for a set of initial conditions in experiments, theoretical model and the normal form equation of subcritical Hopf bifurcation. We find that the critical rate required for tipping is small when the initial condition is closer to the unstable limit cycle. We can further notice that subcritical pitchfork bifurcation (with a fifth order nonlinear term) will have the same critical rate for tipping as in the case of subcritical Hopf bifurcation. Thus, we illustrate that preconditioned rate induced tipping can potentially be observed in a wide range of systems exhibiting subcritical Hopf and subcritical pitchfork bifurcations.

## Methods

### Model parameters

The parameters used in numerical experiments are as follows: *j* = (1, …, *N*), *N* = 10, *x*_*f*_ = 0.25, *γ* = 1.4, *M* = 0.01, *ω*_*j*_ = *jπ*, *τ* = 0.2, ${\zeta }_{j}=\frac{1}{2\pi }[{c}_{1}\frac{{\omega }_{j}}{{\omega }_{1}}+{c}_{2}\sqrt{\frac{{\omega }_{1}}{{\omega }_{j}}}]$, where *c*_1_ = 0.1 and *c*_2_ = 0.06 are constant coefficients, *dt* (step size for computation) = 0.01. For demonstrating preconditioned rate induced tipping (Fig. 3d), the initial conditions chosen are *η*_1_(0) = 0.42, ${\dot{\eta }}_{1}(0)=0.55$, *η*_*j*,*j* ≠ 1_(0) = 0 and ${\dot{\eta }}_{j,j\ne 1}(0)=0$. The presence of time delay in the model demands additional initial values for *η*_*j*_ and ${\dot{\eta }}_{j}$ during the time interval *t* ∈ [−*τ*, 0). We set *η*_*j*_ = 0 and ${\dot{\eta }}_{j}=0$ in the range *t* ∈ [−*τ*, 0) for *j* = 1, …, *N*.

In experiments, we estimated the standard deviations of *K*_*H*_ = 31 W and *K*_*f*_ = 23.1 W by repeating the bifurcation analysis 20 times. The hysteresis width (*K*_*H*_ − *K*_*f*_) has a standard deviation of 16.4 W.

## Initial condition in model and experiments

In experiments and in the model, we try to provide similar initial conditions although the method we follow to obtain the desired initial state is different. In the model, we initialize the physical variables *P*′ and *u*′ through the modes *η*_*j*_ and ${\dot{\eta }}_{j}$ as the set of governing equations are in terms of these modes (equation 5). In this study, we provide non-zero initial conditions to the first mode alone i.e., ${\eta }_{1}(0)\ne 0$, ${\dot{\eta }}_{1}(0)\ne 0$, ${\eta }_{j,j\ne 1}(0)=0$ and ${\dot{\eta }}_{j,j\ne 1}(0)=0$. Therefore, the initial condition in velocity and pressure fluctuations can be written as $u^{\prime} (x,0)={\eta }_{1}(0)\cos \,\pi x$ and $p^{\prime} (x,0)=-\frac{\gamma M}{\pi }{\dot{\eta }}_{1}(0)\sin \,\pi x$. In experiments, we use loudspeakers to evolve the system to the desired initial state. Our aim is to establish in experiments, the standing wave corresponding to the first mode, which is the initial condition used in model. The loudspeaker directly provides an excitation in the physical variables *u*′ and *p*′ instead of ${\dot{\eta }}_{j}$ or *n*_*j*_. The input we provide in the physical variable will be distributed among the various modes. In order to efficiently excite the first mode (${\dot{\eta }}_{1}$, *η*_1_), we provide a sinusoidal perturbation with frequency corresponding to the first mode. We provide this perturbation for a finite amount of time so that the standing wave can be established in the duct, and then we turn off the loudspeakers.

## Procedure to estimate the critical rate

### Experiments

In experiments, we maintain the heater power constant at $\tilde{K}=-0.135$ which is within the bistable zone. Then we proceed to find the unstable limit cycle amplitude at that parameter. We excite the system with a sinusoidal perturbation (at the frequency corresponding to the first mode) to establish the initial condition. Once the initial condition is established, we switch off the loudspeakers and check if the finite amplitude perturbation decays in time. We chose a set of initial conditions such that the system remains in the basin of attraction of the fixed point. We increased the heater power from $\tilde{K}=-0.135$ to −0.07 without initial excitation and ensured that the system is within the basin of attraction of the fixed point. Thus, we ascertained that the initial and final parameter values lie within the bistable zone. Then, for each given initial condition, we varied the parameter from $\tilde{K}=-0.135$ to −0.07 at different rates to find the critical rate.

### Theoretical model

In model, the parameter is varied from ${\tilde{k}}_{0}=-0.145$ following $\tilde{k}={\tilde{k}}_{0}+\alpha (t-{t}_{0})$, where *t*_0_ = 50, till ${\tilde{k}}_{e}=-0.064$. For *t* < *t*_0_, we set ${\tilde{k}}_{0}=-0.145$. We set the initial condition *η*_1_(0) = 0.42 and varied ${\dot{\eta }}_{1}(0)$ from 0.39 to 0.565. The other parameters are maintained the same as described in Model parameters (above). Once $\tilde{k}=-0.064$ is reached, we allow the system to evolve (for sufficient time) at this parameter and check if the system reaches the limit cycle or decays to the fixed point. The lowest *α* for which tipping occurs is identified as *c*_*r*_.

### Normal form equation of subcritical Hopf bifurcation

In the normal form equation, $\dot{r}=\mu r+a{r}^{3}-b{r}^{5}$, with *a* = 1, *b* = 1, the parameter *μ* is varied in time as *μ* = *μ*_0_ + *ct* till we reach *μ*_*e*_ = −0.05. The condition for tipping to occur is that the value of *r* at end of the evolution must be greater than the unstable limit cycle amplitude at *μ*_*e*_. The minimum rate at which this condition is satisfied is the critical rate.

### Data acquisition

We used piezoelectric transducers (PCB piezotronics, PCB103B02) having a sensitivity of 217.5 mV/kPa and resolution of 0.2 Pa, to record the pressure fluctuations. A data acquisition system (PCI 6221) acquires the output from the transducer. The transducer is mounted at 325 mm from the end open to the atmosphere. We used four loud speakers (Ahuja AU 60) to provide the initial finite amplitude perturbation. These loudspeakers are mounted at 625 mm from the end of the duct that is open to atmosphere. The signal is created in LabVIEW software and then input to the loud speakers through an amplifier. A DC power supply (TDK-Lambda, GEN 8–400, 0–8 V, 0–400 A) is used to provide the input power to heat the wire mesh. The uncertainty associated with the heater power is 0.4 W. The uncertainty associated with the air flow rate measurement is ±2.1%.

## Electronic supplementary material

Supplementary material

## References

[1] Lewontin, R. C. *The Meaning of Stability* (Springfield, VA, 1969). 5372787

[2] Scheffer M (2001). Catastrophic shifts in ecosystems. Nature.

[3] May RM, Levin SA, Sugihara G (2008). Complex systems: Ecology for bankers. Nature.

[4] Lenton TM (2008). Tipping elements in the earth’s climate system. Proc. Natl. Acad. Sci. USA.

[5] Scheffer, M. *Catastrophic Transitions in Nature and Society*. (Princeton Univ. Press, 2009).

[6] K´efi S (2007). Spatial vegetation patterns and imminent desertification in mediterranean arid ecosystems. Nature.

[7] Jackson JBC (2001). Historical overfishing and the recent collapse of coastal ecosystems. Science.

[8] Scheffer M (2009). Early-warning signals for critical transitions. Nature.

[9] Ashwin P, Wieczorek S, Vitolo R, Cox P (2012). Tipping points in open systems: bifurcation, noise-induced and rate-dependent examples in the climate systems. Philos. Trans. R. Soc. A.

[10] Thompson J, Sieber J (2011). Predicting climate tipping as a noisy bifurcation: A review. Intl. J. Bifurcation Chaos.

[11] Mannella R, Moss F, McClintock PVE (1987). Postponed bifurcations of a ring-laser model with a swept parameter and additive colored noise. Phys. Rev. A..

[12] Erneux T, Laplante JP (1989). Jump transition to a time-independent bifurcation parameter in the bistable ioadate-arsenous acid reaction. J. Chem. Phys..

[13] Mandel P, Erneux T (1984). Laser Lorenz equations with a time-dependent parameter. Phys. Rev. Lett..

[14] Mandel P, Erneux T (1987). The slow passage through a steady bifurcation: delay and memory effects. J. Statistical Phys..

[15] Baer SM, Erneux T, Rinzel J (1987). The slow passage through a Hopf bifurcation: delay, memory effects and resonance. SIAM J. Appl. Math..

[16] Majumdar A, Ockendon J, Howell P, Surovyatkina E (2013). Transitions through critical temperatures in nematic liquid crystals. Phys. Rev. E..

[17] Wieczorek S, Ashwin P, Luke CM, Cox PM (2011). Excitability in ramped systems: the compost-bomb instability. Proc. R. Soc. A.

[18] Subramanian P, Mariappan S, Sujith RI, Wahi P (2010). Bifurcation analysis of thermoacoustic instability in a horizontal rijke tube. Intl. J. Spray Combust. Dyn.

[19] Gopalakrishnan EA, Sujith RI (2014). Influence of system parameters on the hysteresis characteristics of a horizontal Rijke tube. Intl. J. Spray Combust. Dyn..

[20] Lieuwen, T. C. *Unsteady Combustor Physics* (Cambridge University Press, 2012).

[21] Rayleigh JWS (1878). The explanation of certain acoustical phenomena. Nature.

[22] Fisher, S. C. & Rahman, S. A. Remembering the giants: Apollo rocket propulsion development in *NASA monographs in Aerospace History series* (NASA History Division, Washington, DC, 2009).

[23] Balasubramanian K, Sujith RI (2008). Thermoacoustic instability in a Rijke tube: Non-normality and nonlinearity. Phys. Fluids..

[24] Gopalakrishnan EA (2016). Early warning signals for critical transitions in a thermoacoustic system. Scientific Reports.

[25] Meirovitch, L. *Analytical Methods in Vibrations* (Macmillan, New York, 1967).

[26] Zinn BT, Lores ME (1971). Application of the Galerkin method in the solution of non-linear axial combustion instability problems in liquid rockets. Combust. Sci. Technol..

