# Supplementary material for: Experimental investigation on preconditioned rate induced tipping in a thermoacoustic system
Source: Sci Rep. 2017 Jul 14;7:5414. doi: 10.1038/s41598-017-05814-y (PMC5511272; doi:10.1038/s41598-017-05814-y)
Supplement: Supplementary file 1 — Supplementary material [file 41598_2017_5814_MOESM1_ESM.pdf]

Supplementary Information

Experimental investigation on preconditioned rate-induced tipping in a thermoacoustic system

Tony, J.[1], Subarna, S[1]., Syamkumar, K. S.[1], Sudha, G.[1], Akshay, S.[1], Gopalakrishnan E. A.[2], Surovyatkina, E.[3,4] & Sujith R. I.[1]

[1]Indian Institute of Technology Madras, India.
[2]Amrita School of Engineering, Coimbatore, India.
[3]Space Research Institute of Russian Academy of Sciences, Moscow, Russia
[4]Potsdam Institute for Climate Impact Research, Germany.

Supplementary Note 1. Preconditioned rate induced tipping in the normal form equation of subcritical Hopf bifurcation

We, here, show in the normal form equation of subcritical Hopf bifurcation (equation 1), that for an arbitrary initial condition, one can find a sufficiently large rate of change of the control parameter such that the system tips. We then show that for the same initial condition, there exists small enough rates for which the system does not tip. This, therefore, demonstrates that tipping can occur in the system solely due to fast enough variation of the control parameter.

The normal form equation is given as

$$\dot{r} = \mu r + a r^3 - b r^5, \qquad \dot{\theta} = 1 \tag{1}$$

where a and b are fixed positive numbers. The Hopf point of the system corresponds to $\mu_{\mathrm{H}} = 0$, and the fold point to $\mu_{\mathrm{F}} = -a^2/4b$. Hence, the parameter values in the region (μ_{F}, μ_{H}) comprise the bistable zone. The system has two attractors - a stable fixed point corresponding to $r = 0$ and a stable limit cycle corresponding to $r = r_s$. These are separated by a repellor - an unstable limit cycle at $r = r_u$.

$$r_s,\ r_u = \sqrt{\frac{a}{2b} \pm \frac{1}{2b}\sqrt{a^2 + 4\mu b}}, \qquad \mu \in (-a^2/4b\,, 0) \tag{2}$$

We start with an initial parameter value μ_0 in the bistable region, and with an initial amplitude r_0 such that the system is in the basin of attraction of the fixed point. Say, now we vary the parameter with a fixed rate $\dot{\mu} = c$ ($c > 0$). Then, there is a possibility for the system to undergo preconditioned rate induced tipping. However, before going over to that, we would like to make some details clear. The points $r = r_u$ and $r = r_s$ no longer correspond to limit cycles (because the system cannot stay on any of these points as $\dot{\mu} \neq 0$). However, these points still demarcate the basins of attraction in the system at any time instant - trajectories move away from $r = r_u$ either towards $r = 0$ or towards $r = r_s$ in the bistable zone. And since μ itself is varying, the points $r = r_u$ and $r = r_s$ change with time. We, nevertheless, use the terms stable and unstable limit cycle loosely, for the purpose of explanation.

Now, if $\dot{\mu} = 0$, the parameter must be beyond the Hopf point for the system to tip to the stable limit cycle. However, for $\dot{\mu} \neq 0$, one can have tipping before the system reaches the Hopf point, just due to fast variation of μ. The following discussion explains the mechanism for such a tipping.

We can see from equation 2 that the unstable limit cycle amplitude r_u decreases with increase in μ. Then, if there exists a rate ($\dot{\mu}$) large enough such that after time t, the decrease in r_u is greater than the decay of the system towards the fixed point, the system has crossed over from the basin of attraction of the fixed point to that of the stable limit cycle. If this situation occurs before μ reaches the Hopf point, we say that the system has undergone preconditioned rate induced tipping. To express this mathematically, we fix the final value of μ at $\mu_{\mathrm{e}} < \mu_{\mathrm{H}}$ (and $\mu_{\mathrm{e}} > \mu_0$),

and label the corresponding unstable limit cycle amplitude as r_{u_e}. This condition can then be expressed as

$$r(t) > r_u(t); \quad \mu(t) \leq \mu_\mathrm{e} \tag{3}$$

To show that tipping can occur for some rate $\dot{\mu} = c$, one would substitute in the above equation

$$r(t) = r_0 + \int_0^t (\mu r + ar^3 - br^5)\, dt \tag{4}$$

This, however, is of little use as it is difficult to integrate. We circumvent this problem by using the following reasoning. The minima of $\dot{r}$ occurs at

$$\bar{r} = \sqrt{\frac{3a}{10b} - \frac{1}{10b}\sqrt{9a^2 + 20\mu b}} \tag{5}$$

This represents the maximum possible velocity towards the fixed point ($r = 0$) at any instant of time. If we can find a rate c_{th} such that, even if the system were to attain this maximum velocity at every time instant, the system tips, then for any $c > c_{th}$, the system will always tip. In other words, we would have confirmed that the system can undergo preconditioned rate induced tipping for sufficiently large rates. We note here that the threshold rate (c_{th}) is not the critical rate (critical rate is the smallest rate above which the system tips). The existence of a threshold rate just serves to show that large enough rates ($c > c_{th}$) can tip the system.

We proceed to find this threshold rate. Clearly the parameter at any time instant is given as

$$\mu(t) = \mu_0 + ct \tag{6}$$

Using equations 4, 5 and 6 in equation 3, in the limiting condition ($\mu = \mu_\mathrm{e}$), we have,

$$r_0 + \int_0^{(\mu_e - \mu_0)/c} ((\mu_0 + ct)\bar{r} + a\bar{r}^3 - b\bar{r}^5)\, dt \ > \ r_u((\mu_e - \mu_0)/c) \quad (7)$$

This can now be integrated to obtain[a]

$$r_0 - \frac{\bar{\delta}}{\gamma} > r_{u_e} \quad (8)$$

where,

$$\bar{\delta} = \left\{ 4\mu_0 \left[\frac{\left(\alpha - \sqrt{\beta + \gamma t}\right)^{5/2}}{5} - \frac{\alpha\left(\alpha - \sqrt{\beta + \gamma t}\right)^{3/2}}{3} \right] \right.$$

$$+ \frac{4b}{63}\left(\alpha - \sqrt{\beta + \gamma t}\right)^{1/2}\left[-16\alpha^4 - 8\alpha^3\sqrt{\beta + \gamma t} + 6\alpha^2(6\beta - \gamma t)\right.$$

$$\left. + \alpha(16\beta - 5\gamma t)\sqrt{\beta + \gamma t} + 7(-4\beta^2 + \beta\gamma t + 5\gamma^2 t^2)\right]$$

$$- \frac{4a}{35}\left(\alpha - \sqrt{\beta + \gamma t}\right)^{5/2}\left(2\alpha + 5\sqrt{\beta + \gamma t}\right)$$

$$\left. + \frac{4b}{63}\left(\alpha - \sqrt{\beta + \gamma t}\right)^{7/2}\left(2\alpha + 7\sqrt{\beta + \gamma t}\right) \right\}_{(\mu_e - \mu_0)/c}^{0} \quad (9)$$

and

$$\alpha = \frac{3a}{10b}; \ \beta = \frac{9a^2}{100b^2} + \frac{\mu_0}{5b}; \ \gamma = \frac{c}{5b} \quad (10)$$

Now, we can notice that for the integration limit $t = 0$, $\gamma t = 0$, and for $t = (\mu_e - \mu_0)/c$, $\gamma t = (\mu_e - \mu_0)/5b$. Thus $\bar{\delta}$ does not depend on c. Further, $\bar{\delta} > 0$ because the integrand in

[a] The integration has been performed using free online version of Wolfram Alpha (http://www.wolframalpha.com/calculators/integral-calculator/) and the result has been cross-checked with Online Integral Calculator (http://integral-calculator.com/)

equation 7 is always negative in the region of integration. Thus, from equation 8 we obtain a threshold rate

$$c_{th} = \frac{5b\bar{\delta}}{\left(r_0 - r_{u_e}\right)} \tag{11}$$

and for $c > c_{th}$, we have preconditioned rate induced tipping. Of course, here we assume that $r_0 > r_{u_e}$. If not, the inequality as well as the sign must be changed while obtaining equation 11 (i.e., $c < -|c_{th}|$) and we will subsequently obtain a contradiction with the choice of $c > 0$. This observation is consistent, because we cannot have tipping if $r_0 < r_{u_e}$, as $\dot{r} < 0$ in the basin of attraction of the fixed point inside the bistable zone. For $r_0 = r_{u_e}$, we require infinite rate for the system to tip.

Now, we show that for any $r_0 > r_{u_e}$, we can find a sufficiently small rate c, such that the system does not tip. Let $\lambda > 2$ be an arbitrary constant, and say the system takes time T for reaching from r_0 to r_{u_e}/λ when the parameter is fixed at $\mu = \mu_0$. That is

$$\frac{r_{u_e}}{\lambda} = r_o + \int_0^T f(\mu_0, r)dt \tag{12}$$

where $f(\mu_0, r) = \mu_0 r + ar^3 - br^5$. Note that as λ increases, so does T. Now, if the parameter is varied at a rate c, we have $\dot{r} = f(\mu_0, r) + crt$. As mentioned previously, the amplitude decays in the bistable zone as long as the system is in the basin of attraction of the fixed point, and therefore, $r_0 > r$. Hence if we use $\dot{r} = f(\mu_0, r) + cr_0 t$ and show tipping does not occur, it will not occur in the actual system. For this case, after time T we have

$$r(T) = r_o + \int_0^T f(\mu_0, r)dt + \frac{cr_0T^2}{2} = \frac{r_{u_e}}{\lambda} + \frac{cr_0T^2}{2} \tag{13}$$

Now if we choose c such that $cr_0T^2/2 < r_{u_e}/\lambda$, we have $r(T) < 2\,r_{u_e}/\lambda$, and because $\lambda > 2$, $r(T) < r_{u_e}$. To not have preconditioned rate induced tipping, we also need to ensure that at time T the parameter μ does not exceed the end point μ_e. At time T we have

$$\mu(T) = \mu_0 + cT < \mu_0 + \frac{2r_{u_e}}{r_0\lambda T} \tag{14}$$

which should be less than or equal to μ_e. We argue that we can always find a λ large enough so that $2r_{u_e}/r_0\lambda T < \mu_\mathrm{e} - \mu_0$. This is because for $\lambda \to \infty$, the final point the system attains in equation 12 tends to zero, and so $T \to \infty$ (that is, the amplitude r asymptotically reaches the origin). Therefore, we can choose a λ large enough (with $\lambda > 2$) such that the combination λT is large and the required condition of $\mu(T) \le \mu_\mathrm{e}$ is satisfied. Now, because at time T the amplitude has gone below the final unstable limit cycle amplitude r_{u_e} and the parameter has not reached the end point μ_e, the system will not tip. So, we have shown that for any initial condition r_0, we can always find a rate $\left(c < 2r_{u_e}/r_0\lambda T^2\right)$ such that the system does not tip. Note that we choose μ_e to be strictly less than the Hopf point, and the above analysis will not hold for the case when $\mu_\mathrm{e} = \mu_\mathrm{H}$ (since $r_{u_e} = 0$ for $\mu_\mathrm{e} = \mu_\mathrm{H}$).

We discard the scenario $\mu_\mathrm{e} = \mu_\mathrm{H}$, because in this case, the system might tip for any positive rate of change of control parameter. We explain the rationale behind this statement in the following discussion.

For $\mu_e = \mu_H$, the unstable limit cycle amplitude at the end point (r_{u_e}) is zero. And we can see from equation 15 that the rate of change of the unstable limit cycle amplitude tends to negative infinity as we approach the Hopf point (since $r_u(\mu_H) = r_{u_e} = 0$).

$$\dot{r}_u = -c/\left(2r_u\sqrt{a^2 + 4\mu b}\right) \tag{15}$$

Now say for this case, the system has not tipped at a parameter value μ_1 very close to the Hopf point. Hence, the amplitude r_1 is less than r_u at this point, and since r_u close to the Hopf point is small, so is r_1 (i.e., $r_1 < r_u(\mu_1) \ll 1$). Hence, we can linearize the rate of change of r about the fixed point (i.e. about $r = 0$), and correspondingly we get $\dot{r} = \mu r$. Solving this equation, we obtain

$$r(t) = r_1 \exp[(\mu(t)^2 - {\mu_1}^2)/2c] \tag{16}$$

Now, since the value inside exponential in equation (16) is negative (as $\mu_1 < \mu(t) \leq 0$), we see that $0 < r(t) < r_1$, while on the other hand, r_u continuously approaches zero at an infinite rate (equation 15). Therefore, at some parameter value between μ_1 and $\mu_e = \mu_H$, the amplitude r crosses r_u and the system tips. In other words, irrespective of the rate c, the system tips. To avoid such a case, we choose an end point μ_e strictly less than μ_H.

In summary, we have shown that for any initial condition $r_0 > r_{u_e}$, there exist large enough rates for which the system will cross-over to the basin of attraction of the stable limit cycle before reaching the Hopf point. Also, we have shown that for the same initial condition, there are sufficiently small rates for which the system does not exhibit this behavior. This implies that there exists a critical rate above which the system will tip. Therefore, we have shown that the

rate at which the parameter is varied can determine whether the system tips, thus establishing the occurrence of preconditioned rate induced tipping.

Supplementary Note 2. Theoretical model of the thermoacoustic system

We consider a theoretical model[1] that incorporates the feedback that exists between the sound waves and the heat release rate fluctuations. The model is derived from the conservation equations of momentum and energy given as follows[2].

$$\tilde{\rho}\frac{\partial \tilde{u}}{\partial \tilde{t}} + \tilde{\rho}\tilde{u}\frac{\partial \tilde{u}}{\partial \tilde{x}} = -\frac{\partial \tilde{p}}{\partial \tilde{x}}, \tag{17}$$

$$\frac{\partial \tilde{p}}{\partial \tilde{t}} + \tilde{u}\frac{\partial \tilde{p}}{\partial \tilde{x}} + \gamma \tilde{p}\frac{\partial \tilde{u}}{\partial \tilde{x}} = (\gamma - 1)\tilde{\dot{Q}}, \tag{18}$$

where γ is the ratio of specific heats of air, $\tilde{p}$ is pressure, $\tilde{\rho}$ is density, $\tilde{u}$ is velocity and $\tilde{\dot{Q}}$ is the heat release rate per unit volume. The variables in equations (17) and (18) are decomposed into mean and fluctuating variables. In the system considered in this study, the amplitude of the fluctuations are small. Thus, linearized equations are sufficient to describe the propagation of sound in the duct. Hence, terms involving the product of fluctuating variables are neglected. The effects of mean flow (low Mach number approximation[3]) and temperature gradient are also neglected to obtain the following equations.

$$\bar{\rho}\frac{\partial \tilde{u}'}{\partial \tilde{t}} + \frac{\partial \tilde{p}'}{\partial \tilde{x}} = 0 \tag{19}$$

$$\frac{\partial \tilde{p}'}{\partial \tilde{t}} + \gamma \bar{p}\frac{\partial \tilde{u}'}{\partial \tilde{x}} = (\gamma - 1)\tilde{\dot{Q}}'. \tag{20}$$

The heat release rate fluctuations per unit volume ($\tilde{\dot{Q}}'$) is given by[4]:

$$\tilde{\dot{Q}}' = \frac{2L_w(T_w - \bar{T})}{S\sqrt{6}}\sqrt{\pi\lambda C_V \bar{\rho} d_w}\left[\sqrt{\left|\frac{u_0}{3} + \tilde{u}'(\tilde{t} - \tilde{\tau})\right|} - \sqrt{\frac{u_0}{3}}\right]\delta\left(\tilde{x} - \tilde{x}_f\right), \tag{21}$$

Where λ is the heat conductivity of air, C_V is the specific heat at constant volume, u_0 is the mean velocity of the flow, $\bar{\rho}$ and $\bar{T}$ are the mean density and temperature of the flow respectively, S is the cross-sectional area of the duct, L_w is the effective wire length, T_w is the temperature of the wire, d_w is the wire diameter and $\tilde{x}_f$ is the location of the heater. The response of the heat release rate to the sound waves (velocity fluctuations) is nonlinear even though the propagation of sound can be described using linear equations. Further, the term $\tilde{u}'(\tilde{t}-\tilde{\tau})$ in equation (21) represents the time delayed response of the heat release rate to the fluctuating velocity. We express equations (19) and (20) in terms of the following non-dimensional quantities.

$$x = \tilde{x}/L, \qquad t = \tilde{t}c_0/L, \qquad u' = \tilde{u}'/u_0, \qquad p' = \tilde{p}'/\bar{p}, \qquad M = u_0/c_0, \tag{22}$$

where L is the length of the duct, c_0 is the speed of sound in the duct and $\bar{p}$ is the mean pressure. The non-dimensionalized equations of momentum and energy are as follows.

$$\gamma M \frac{\partial u'}{\partial t} + \frac{\partial p'}{\partial x} = 0, \tag{23}$$

$$\frac{\partial p'}{\partial t} + \gamma M \frac{\partial u'}{\partial x} = k\left[\sqrt{\left|\frac{1}{3} + u'(t-\tau)\right|} - \sqrt{\frac{1}{3}}\right]\delta\left(x - x_f\right), \tag{24}$$

where $k = (\gamma - 1)\frac{4(T_w - \bar{T})}{\gamma M \bar{p} c_0 S \sqrt{6}}\sqrt{\pi\lambda C_V \bar{\rho} d_w u_0}$.

Then, we expand the velocity and pressure fluctuations in terms of the spatial modes of the duct.

$$u' = \sum_{j=1}^{N} \eta_j \cos j\pi x, \qquad p' = -\sum_{j=1}^{N} \frac{\gamma M}{j\pi} \dot{\eta}_j \sin j\pi x \tag{25}$$

where η_j and $\dot{\eta}_j$ are time varying quantities. The above expansion for u' and p' can be substituted in equations (23) and (24) to convert the partial differential equations to the following set of ODEs.

$$\ddot{\eta}_j + 2\zeta_j\omega_j\dot{\eta}_j + \omega_j^2\eta_j = -kj\pi\left[\sqrt{\left|\frac{1}{3} + u_f'(t-\tau)\right|} - \sqrt{\frac{1}{3}}\right]\sin j\pi x_f \tag{26}$$

We introduce a damping term[5] with damping coefficient $\zeta_j = \frac{1}{2\pi}\left[c_1\frac{\omega_j}{\omega_1} + c_2\sqrt{\frac{\omega_1}{\omega_j}}\right]$, where c_1 and c_2 are constant coefficients, in equation (26). The non-dimensional angular frequency of the jth mode is given by $\omega_j = j\pi$.

Supplementary Note 3. Identifying initial conditions for observing preconditioned rate induced tipping in experiments and in the model

In this section, we identify initial conditions that are required to observe preconditioned rate induced tipping in experiments and model. The initial conditions should be such that the system is within the basin of attraction of the fixed point in the bistable region. If the system decays to the fixed point, we can conclude that the initial condition is within the basin of attraction of the fixed point. Then, we use this initial condition to observe preconditioned rate induced tipping in the system.

In experiments, we maintained the normalized heater power at $\widetilde{K}_0 = -0.135$ (in the bistable region) and provided a finite amplitude excitation using loudspeakers. We find that the perturbation decays as illustrated in Supplementary Fig. 3.1. Similarly, in the model, we identified initial conditions in $\tilde{k}$ and p' such that the system is in the basin of attraction of the fixed point in the bistable zone (see Supplementary Fig. 3.2). We maintained these initial conditions in experiments and in the model to identify preconditioned rate induced tipping.

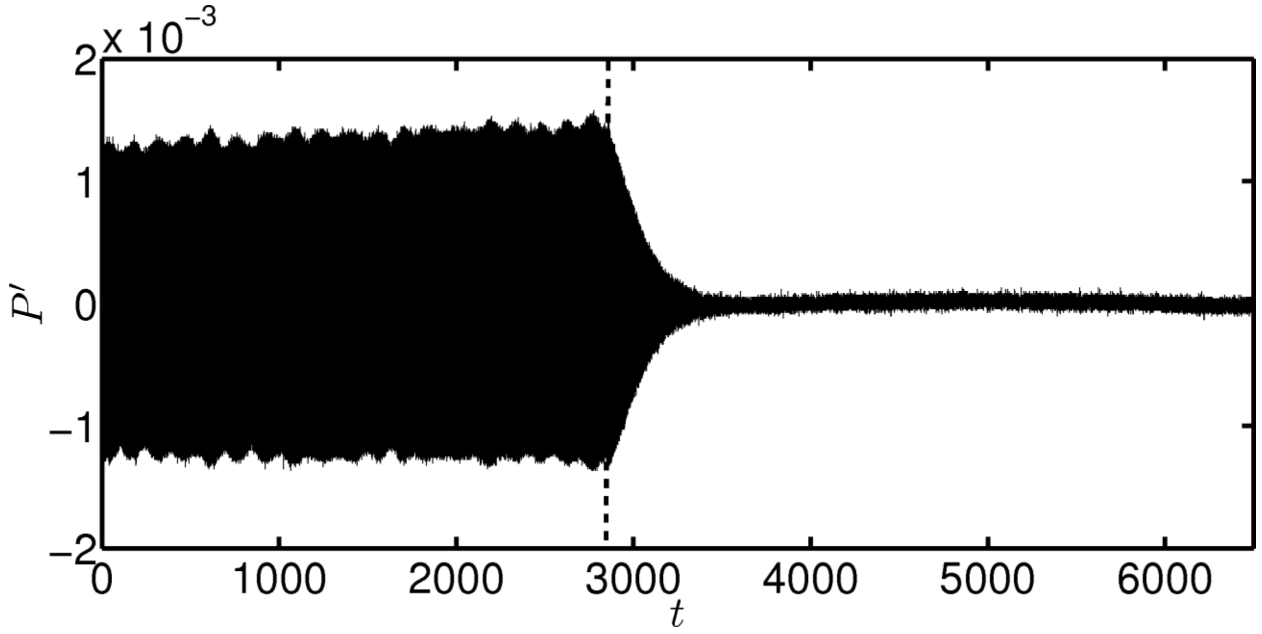

Supplementary Figure 3.1. Plot depicting the decay of a finite amplitude perturbation when the system is within the basin of attraction of the fixed point. The decay of perturbation happens as the amplitude of initial excitation is less than the unstable limit cycle amplitude. The vertical dashed line represents the time stamp when the loud speaker is switched off.

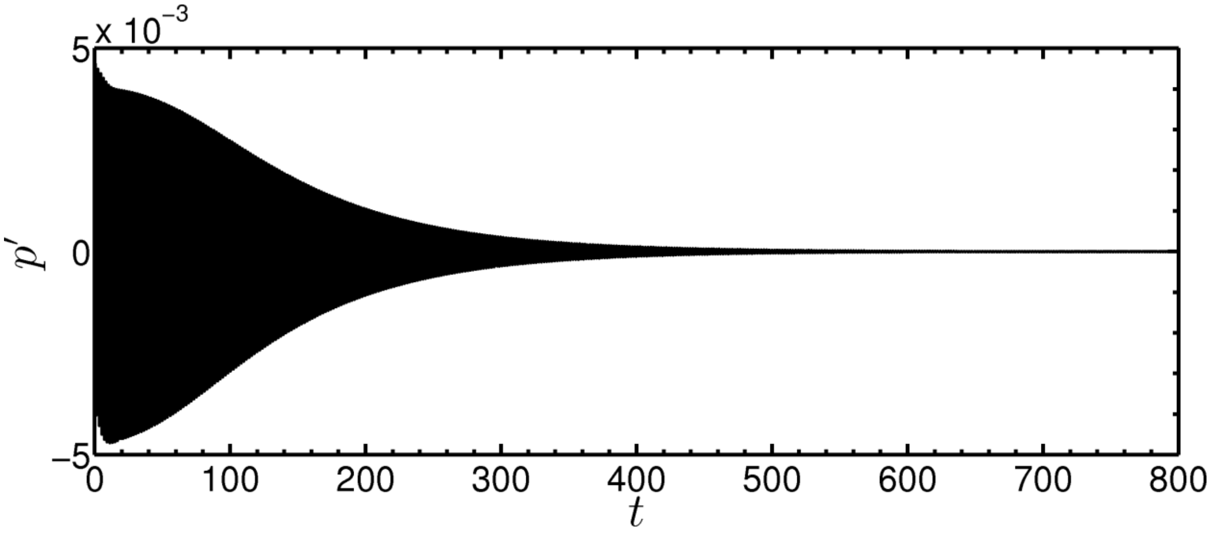

Supplementary Figure 3.2. **Plot depicting the decay of a finite amplitude perturbation in the theoretical model**. The control parameter is maintained at $\tilde{k}_0 = -0.145$, which is within the bistable region. The initial conditions, $\eta_1(0) = 0.42$, $\dot{\eta}_1(0) = 0.55$, $\eta_{j,j\neq1}(0) = 0$ and $\dot{\eta}_{j,j\neq1}(0) = 0$, are chosen such that p' decays to zero. This initial condition places the system between the unstable limit cycle and the stable fixed point.

Now, we demonstrate that the critical rate for preconditioned rate induced tipping is a function of the initial conditions. In order to show this, we repeat the same numerical experiment as in Figs. 3 (c) & (d) (in the main text) but with a slightly different initial condition. We set the value of $\dot{\eta}_1(0)$ to 0.53. The evolution of p' in time for this set of parameters and initial conditions is shown in Supplementary Fig. 3.3. The system eventually decays to the fixed point when $\tilde{k}$ is varied at the rate $\alpha = 1.29\times10^{-3}$ (same rate as in Figs. 3 (c) & (d)). This means that this value of α is below the critical rate for this set of initial conditions. The parameter should be varied at a higher rate to observe tipping in this case.

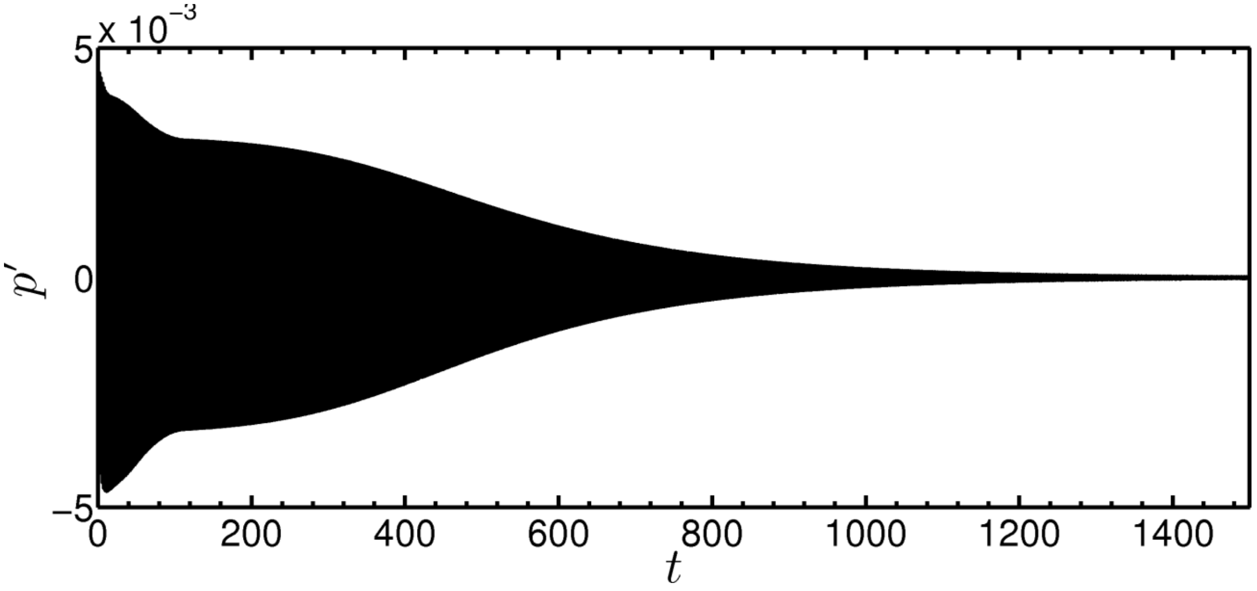

Supplementary Figure 3.3. **Evidence for the dependence of critical rate on the initial conditions in the theoretical model**. The control parameter is maintained at $\tilde{k}_0 = -0.145$ until $t_0 = 50$. Then $\tilde{k}$ is increased to -0.064 according to the relation $\tilde{k} = \tilde{k}_0 + \alpha(t - t_0)$, where $\alpha = 1.29\times10^{-3}$. The initial conditions are $\eta_1(0) = 0.42$, $\dot{\eta}_1(0) = 0.53$, $\eta_{j,j\neq1}(0) = 0$ and $\dot{\eta}_{j,j\neq1}(0) = 0$. The parameters are the same as in Fig. 3 except for a slightly different value for $\dot{\eta}_1(0)$. The initial perturbation decays since the critical rate for tipping is higher than the present value of α.

Supplementary References

1. Balasubramanian, K. & Sujith, R. I. Thermoacoustic instability in a Rijke tube: Non-normality and nonlinearity. *Phys. Fluids.* **20**, 044103 (2008).

2. Kundu, P. K. & Cohen, I. M. *Fluid Mechanics, 3rd edition* (Academic Press, 2004).

3. Nicoud, F. & Wieczorek, K. About the zero Mach number assumption in the calculation of thermoacoustic instabilities. *Intl. J. Spray Combust. Dyn.* **1**, 67 (2009).

4. Heckl, M. A. Active control of the noise from a Rijke tube. *J. Sound. Vib.* **124**, 117–133 (1988).

5. Matveev K. I. *Thermoacoustic instabilities in the Rijke tube: experiments and modeling*. PhD thesis, California Institute of Technology (2011).
